# Supplementary material for: A safe, T cell-inducing heterologous vaccine against elephant endotheliotropic herpesvirus in a proof-of-concept study
Source: Nat Commun. 2025 Oct 3;16:8374. doi: 10.1038/s41467-025-64004-x (PMC12494954; doi:10.1038/s41467-025-64004-x)
Supplement: Supplementary file 1 — Supplementary Information [file 41467_2025_64004_MOESM1_ESM.pdf]

Supplementary Information for

A safe, T cell-inducing heterologous vaccine against elephant endotheliotropic herpesvirus in a proof-of-concept study

Tanja Maehr, Javier Lopez, Gabby Drake, Frederico M. Ferreira, Richard Fraser, Rebecca Mckown, Reshma Kailath, Susan Morris, Adam Chambers, Leo P. Graves, Susan L. Walker, Akbar Dastjerdi, Katie L. Edwards, Helder I. Nakaya, Falko Steinbach

*Corresponding authors:
Falko Steinbach: f.steinbach@surrey.ac.uk
Tanja Maehr: tanja.maehr@apha.gov.uk

This Supplementary Information includes the following Supplementary Figures and Tables:

Supplementary Fig. 1: Schematic representation of pre- and post-vaccination unstimulated and antigen-stimulated whole blood RNA samples used to generate contrasts for systems immunology analysis.

Supplementary Fig. 2: Western blot analysis of elephant plasma to detect antibodies against vaccine antigens EE2 and major capsid protein (MCP).

Supplementary Fig. 3: Amino acid sequence identity (%) of elephant endotheliotropic herpesvirus 1A (EEHV-1A) vaccine antigens EE2 and major capsid protein (MCP) with homologous proteins from EEHV genotypes affecting Asian elephants.

Supplementary Table 1 Primer sequences used to generate recombinant modified vaccinia Ankara virus rMVA-EE2-MCP

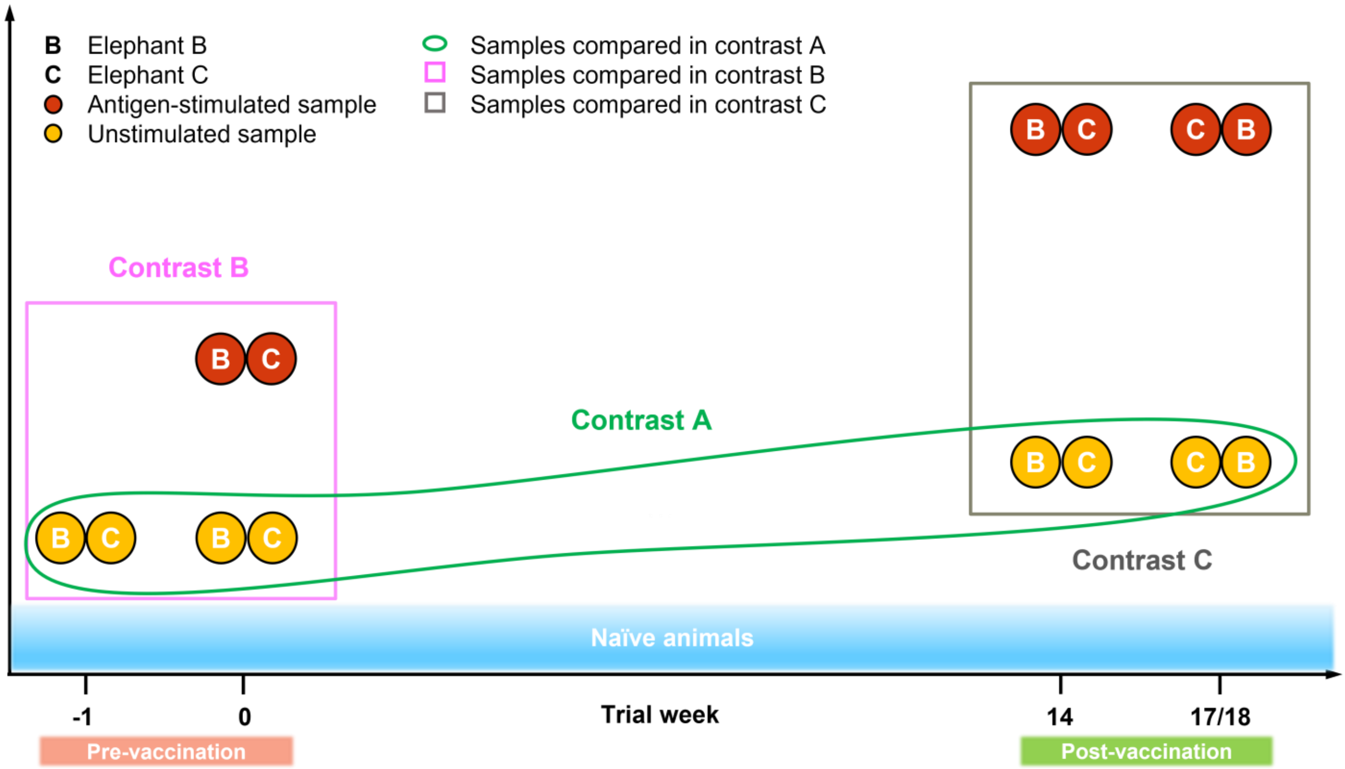

Supplementary Fig. 1: Schematic representation of pre- and post-vaccination unstimulated and antigen-stimulated whole blood RNA samples used to generate contrasts for systems immunology analysis. Blood samples were collected from two elephants (B and C; n = 2) at multiple time points. Yellow circles represent unstimulated samples, and red circles represent stimulated samples. The boxes and the ellipse around samples indicate the sets of samples used to generate transcriptomic comparisons: contrast A (green ellipse) compares unstimulated samples before and after vaccination; contrast B (pink box) compares pre-vaccination samples stimulated with vaccine antigens against unstimulated pre-vaccination samples; and contrast C (grey box) compares post-vaccination samples stimulated with vaccine antigens against unstimulated post-vaccination samples. The blue shaded area indicates the expected baseline antigen response in immunologically naïve animals, whereas the adult elephants in this study exhibit background immunity consistent with prior elephant endotheliotropic herpesvirus exposure, as indicated by baseline *IFNG* gene expression in response to antigen stimulation (see Fig. 2, main text).

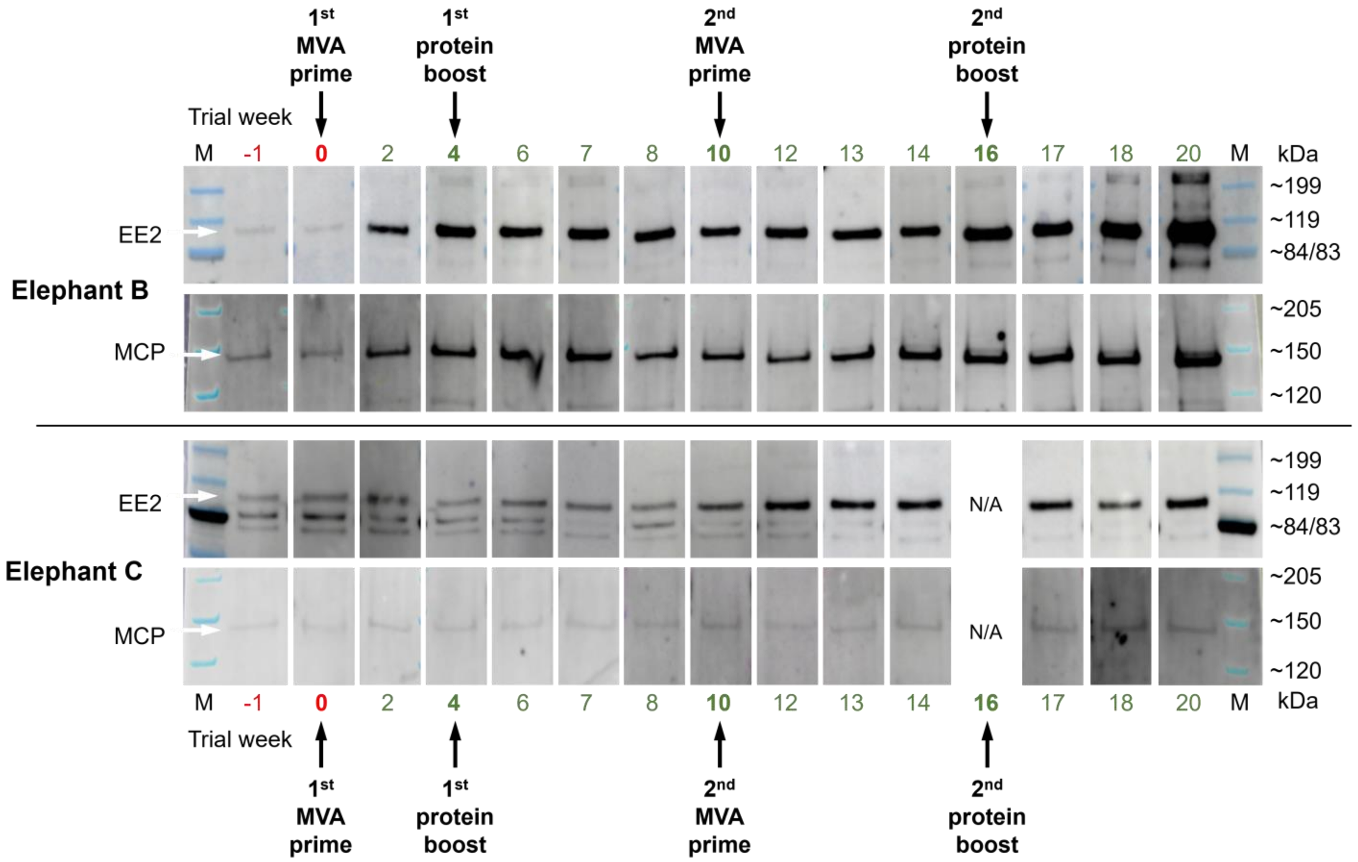

Supplementary Fig. 2: Western blot analysis of elephant plasma to detect antibodies against vaccine antigens EE2 and major capsid protein (MCP). Western blot probing of plasma samples against recombinant (r) EE2 and rMCP was performed to monitor antibody presence before and during prime-boost vaccination courses. As shown for elephants B and C, which participated in stage II of the proof-of-concept study (*n* = 2), antibodies against both antigens were present at near-detection levels prior to vaccination but generally increased throughout the immunisation period. These findings reflect latent infection as well as serological responses to prime-boost vaccinations. N/A – not available: plasma samples could not be obtained for elephant C in trial week 16; plasma samples were also not obtained in trial week 3 of both animals; M – molecular weight markers: iBright Prestained Protein Ladder (10–199 kDa) for EE2 panels, Spectra Multicolor High Range Protein Ladder (40–270 kDa) for MCP panels. Western blots were performed for the two biological replicates (*n* = 2) described above. Uncropped blots in Source Data file.

a

Amino acid identity (%) for the EE2 protein							Viruses
1	2	3	4	5	6		
	98.4	99.2	98.3	60.1	60.5	1	NC_020474.2_EEHV1A
		98.1	98.5	59.9	60.3	2	KC618527.1_EEHV1A
			98.0	59.8	60.2	3	KC462164.1_EEHV1B
				59.8	60.2	4	JX011082.3_EEHV1B
					99.6	5	NC_024696.1_EEHV5
						6	PP906086.1_EEHV5

b

Amino acid identity (%) for the major capsid protein								Viruses
1	2	3	4	5	6	7		
	99.9	98.9	99.0	70.4	88.5	88.4	1	NC_020474.2_EEHV1A
		98.9	99.0	70.5	88.5	88.4	2	KC618527.1_EEHV1A
			99.9	70.8	88.3	88.2	3	KC462164.1_EEHV1B
				70.7	88.4	88.4	4	AF322977.2_EEHV1B
					70.4	70.3	5	NC_028379.1_EEHV4
						99.6	6	NC_024696.1_EEHV5
							7	PP906086.1_EEHV5

Supplementary Fig. 3: Amino acid sequence identity (%) of elephant endotheliotropic herpesvirus 1A (EEHV-1A) vaccine antigens EE2 and major capsid protein (MCP) with homologous proteins from EEHV genotypes affecting Asian elephants. Nucleotide sequences for EE2 (a) and MCP (b) were retrieved from GenBank (https://www.ncbi.nlm.nih.gov/nuccore/) for EEHV-1A (NC_020474.2, KC618527.1), EEHV-1B (KC462164.1, JX011082.3 or AF322977.2), EEHV-4 (NC_028379.1) and EEHV-5 (NC_024696.1, PP906086.1). These were translated into amino acids and aligned for calculating sequence identities using MegAlign (version 15, Lasergene package, DNASTAR). The resulting identity percentages are shown.

Supplementary Table 1 Primer sequences used to generate recombinant modified vaccinia Ankara virus rMVA-EE2-MCP

Primer name	Forward (F) primer sequence (5′ to 3′)	Reverse (R) primer sequence (5′ to 3′)
B8 flinv	ATTTCTCGGTAGCACATC	GGTGTTGTTTGTTATTTGAC
F11 flinv	ATTGGGGCTTTTTGTACAATAAATGG	AGTGTAACAACCAACGATAAAAATAATATTG
EE2	TGTCTCTGCGCTACACTACG	CCTCTTCAGTCTTCTCACATCG
B8FI	CAACGCAGAGGTCACACG	CCTCTTCAGTCTTCTCACATCG
MCP	TGGATGACATCACCATCTCG	TCAATGATAGTTCCTGACCATCC
F11FI	CGCGTTGTGTAAAGTTACCG	TCAATGATAGTTCCTGACCATCC
Purity	TCAATGATAGTTCCTGACCATCC	CGGCGAGTTCATCTACAAGG
