# Supplementary material for: A safe, T cell-inducing heterologous vaccine against elephant endotheliotropic herpesvirus in a proof-of-concept study
Source: Nat Commun. 2025 Oct 3;16:8374. doi: 10.1038/s41467-025-64004-x (PMC12494954; doi:10.1038/s41467-025-64004-x)
Supplement: Supplementary file 2 — Description of Additional Supplementary Files [file 41467_2025_64004_MOESM2_ESM.pdf]

Description of Additional Supplementary Files

Supplementary Data 1 (a-c): Differential gene expression in whole blood before and after vaccination.

Supplementary Data 2 (a, b): Gene Ontology (GO) over-representation analysis of differentially expressed genes (DEG) significantly upregulated in response to antigen stimulation before and after vaccination.

Supplementary Data 3 (a-d): Remaining over-represented Gene Ontology (GO) terms following reduction of redundant terms from contrasts comparing unstimulated vs. antigen-stimulated samples before and after vaccination.

Supplementary Data 4 (a, b): Reactome over-representation analysis of differentially expressed genes (DEG) significantly upregulated upon antigen stimulation before and after vaccination.
