# Supplementary material for: A safe, T cell-inducing heterologous vaccine against elephant endotheliotropic herpesvirus in a proof-of-concept study
Source: Nat Commun. 2025 Oct 3;16:8374. doi: 10.1038/s41467-025-64004-x (PMC12494954; doi:10.1038/s41467-025-64004-x)
Supplement: Supplementary file 7 — Reporting summary [file 41467_2025_64004_MOESM7_ESM.pdf]

nature portfolio

Corresponding author(s): Falko Steinbach

Last updated by author(s): Jul 24, 2025

# Reporting Summary

Nature Portfolio wishes to improve the reproducibility of the work that we publish. This form provides structure for consistency and transparency in reporting. For further information on Nature Portfolio policies, see our Editorial Policies and the Editorial Policy Checklist.

## Statistics

For all statistical analyses, confirm that the following items are present in the figure legend, table legend, main text, or Methods section.

| n/a | Confirmed | |
|---|---|---|
| ☐ | ☒ | The exact sample size ($n$) for each experimental group/condition, given as a discrete number and unit of measurement |
| ☐ | ☒ | A statement on whether measurements were taken from distinct samples or whether the same sample was measured repeatedly |
| ☐ | ☒ | The statistical test(s) used AND whether they are one- or two-sided<br>*Only common tests should be described solely by name; describe more complex techniques in the Methods section.* |
| ☒ | ☐ | A description of all covariates tested |
| ☐ | ☒ | A description of any assumptions or corrections, such as tests of normality and adjustment for multiple comparisons |
| ☒ | ☐ | A full description of the statistical parameters including central tendency (e.g. means) or other basic estimates (e.g. regression coefficient) AND variation (e.g. standard deviation) or associated estimates of uncertainty (e.g. confidence intervals) |
| ☐ | ☒ | For null hypothesis testing, the test statistic (e.g. $F$, $t$, $r$) with confidence intervals, effect sizes, degrees of freedom and $P$ value noted<br>*Give P values as exact values whenever suitable.* |
| ☒ | ☐ | For Bayesian analysis, information on the choice of priors and Markov chain Monte Carlo settings |
| ☒ | ☐ | For hierarchical and complex designs, identification of the appropriate level for tests and full reporting of outcomes |
| ☒ | ☐ | Estimates of effect sizes (e.g. Cohen's $d$, Pearson's $r$), indicating how they were calculated |

*Our web collection on statistics for biologists contains articles on many of the points above.*

## Software and code

Policy information about availability of computer code

| | |
|---|---|
| Data collection | RNA sequencing was performed on a NovaSeq 6000 instrument (Illumina, San Diego, CA, USA). FastQC (v0.11.9) was used for quality checks on sequencing reads. Reads were aligned to the Elephas maximus indicus reference genome (annotation release GCF_024166365.1-RS_2023_02) using the Subread package (v2.0.6) and counted using featureCounts (v2.0.6). Reverse transcription quantitative PCR gene expression data was collected on an AriaMx Real-Time PCR System (Agilent Technologies, Stockport, UK). Western blot membranes were imaged on an Azure c280 digital imager (Cambridge Bioscience, Cambridge, UK). |
| Data analysis | Quality control, normalisation and statistical analyses of RNA sequencing data were conducted using the DESeq2 package (v1.44) in R/ Bioconductor (v4.3.2 and v3.19, respectively). Over-representation analysis was carried out using the Enrichr analysis tool, employing its integrated Human Gene Atlas (original release not versioned; accessed 2024), Gene Ontology Biological Process (GOBP; release 2023) and Reactome (release 2022) databases. The Agilent AriaMx software (v1.8) was used for RT-qPCR gene expression analysis. |

For manuscripts utilizing custom algorithms or software that are central to the research but not yet described in published literature, software must be made available to editors and reviewers. We strongly encourage code deposition in a community repository (e.g. GitHub). See the Nature Portfolio guidelines for submitting code & software for further information.

## Data

Policy information about availability of data

All manuscripts must include a data availability statement. This statement should provide the following information, where applicable:

- Accession codes, unique identifiers, or web links for publicly available datasets
- A description of any restrictions on data availability
- For clinical datasets or third party data, please ensure that the statement adheres to our policy

The RNA-seq data reported in this study were deposited in the NCBI Gene Expression Omnibus Database under accession number GSE297105 [https://www.ncbi.nlm.nih.gov/geo/query/acc.cgi?acc=GSE297105]. All other data related to the findings of this study are available in the Article and its Supplementary Data and Supplementary Information. Source data for RT-qPCR gene expression analysis and unprocessed western blot images are provided in the Source Data file.

## Research involving human participants, their data, or biological material

Policy information about studies with human participants or human data. See also policy information about sex, gender (identity/presentation), and sexual orientation and race, ethnicity and racism.

| | |
|---|---|
| Reporting on sex and gender | N/A |
| Reporting on race, ethnicity, or other socially relevant groupings | N/A |
| Population characteristics | N/A |
| Recruitment | N/A |
| Ethics oversight | N/A |

Note that full information on the approval of the study protocol must also be provided in the manuscript.

# Field-specific reporting

Please select the one below that is the best fit for your research. If you are not sure, read the appropriate sections before making your selection.

☒ Life sciences ☐ Behavioural & social sciences ☐ Ecological, evolutionary & environmental sciences

For a reference copy of the document with all sections, see nature.com/documents/nr-reporting-summary-flat.pdf

# Life sciences study design

All studies must disclose on these points even when the disclosure is negative.

| | |
|---|---|
| Sample size | The study was designed as a proof-of-concept study and conducted as a single trial on a small cohort of animals from a protected species. The sample size was therefore determined by the number of Asian elephants that were available and suitable for inclusion in a study focussed on vaccine safety. Due to the endangered status of the species, protected contact husbandry, and welfare considerations, only a small number of animals could be included. Accordingly, no statistical method was used to predetermine sample size. |
| Data exclusions | No data were excluded from the analyses, except for samples that did not meet downstream quality checks as stated in the manuscript. |
| Replication | Due to the above sample size limitations, biological replication in this trial was limited, but performed. |
| Randomization | Randomization was not possible due to the nature of the study (and the limited availability of animals). |
| Blinding | Blinding was not possible in line with 3R animal review ethics. |

# Reporting for specific materials, systems and methods

We require information from authors about some types of materials, experimental systems and methods used in many studies. Here, indicate whether each material, system or method listed is relevant to your study. If you are not sure if a list item applies to your research, read the appropriate section before selecting a response.

## Materials & experimental systems

| n/a | Involved in the study |
|---|---|
| ☐ | ☒ Antibodies |
| ☐ | ☒ Eukaryotic cell lines |
| ☒ | ☐ Palaeontology and archaeology |
| ☐ | ☒ Animals and other organisms |
| ☒ | ☐ Clinical data |
| ☒ | ☐ Dual use research of concern |
| ☒ | ☐ Plants |

## Methods

| n/a | Involved in the study |
|---|---|
| ☒ | ☐ ChIP-seq |
| ☒ | ☐ Flow cytometry |
| ☒ | ☐ MRI-based neuroimaging |

# Antibodies

**Antibodies used**

Horseradish peroxidase-conjugated Protein A/G was used for western blot detection of plasma antibodies:
Reagent name: Pierce Recombinant Protein A/G, Peroxidase Conjugated
Supplier: Thermo Scientific (Pierce)
Catalogue number: 32490
Lot number: WH324034
Dilution used in western blot assays: 1:5,000

**Validation**

Protein A/G is a recombinant fusion protein used for binding to the Fc region of immunoglobulins. It is used to probe and detect antibodies of many different species hosts in a variety of immunoassays, including western blots, as per the manufacturer's website (https://www.thermofisher.com/order/catalog/product/32490?SID=srch-srp-32490).

The use of Protein A/G for detection of Asian elephant serum antibodies by western blot or ELISA has been demonstrated in the following publications:
1. Paungpin, W. et al. Serosurveillance for pandemic influenza A (H1N1) 2009 virus infection in domestic elephants, Thailand. PLoS One 12, e0186962 (2017).
2. Hoornweg, T. E. et al. Elephant endotheliotropic herpesvirus is omnipresent in elephants in European zoos and an Asian elephant range country. Viruses 13, 283 (2021).
3. Hoornweg, T. E. et al. Young elephants in a large herd maintain high levels of elephant endotheliotropic herpesvirus-specific antibodies and do not succumb to fatal haemorrhagic disease. Transbound. Emerg. Dis. 69, e3379-e3385 (2022).

# Eukaryotic cell lines

Policy information about cell lines and Sex and Gender in Research

**Cell line source(s)**

DF-1: Continuous chicken cell line originally derived from chicken embryonic fibroblasts (Gallus gallus); obtained from the American Type Culture Collection (Manassas, VA, USA).
Sf9: Continuous insect cell line originally derived from pupal ovarian tissue from the fall armyworm (Spodoptera frugiperda); obtained from Fisher Scientific (Loughborough, UK).
Tni High Five: Continuous insect cell line originally derived from ovarian tissue from the cabbage looper (Trichoplusia ni); obtained from Fisher Scientific, UK.

**Authentication**

No authentication was performed for these cell lines as they were obtained from recognised commercial sources or cell repositories.

**Mycoplasma contamination**

All three cell lines were tested for mycoplasma at intervals. At the time of use, they tested negative.

**Commonly misidentified lines**
(See ICLAC register)

There are no commonly misidentified cell lines for all three cell lines used in this study.

# Animals and other research organisms

Policy information about studies involving animals; ARRIVE guidelines recommended for reporting animal research, and Sex and Gender in Research

**Laboratory animals**

This study did not involve laboratory animals.

**Wild animals**

This study did not use wild animals. Adult Asian elephants (Elephas maximus) housed at a zoological collection were used.

**Reporting on sex**

The sex of the study animals is stated in the manuscript. Data are not disaggregated by sex, as this was not applicable to the study objectives in this proof-of-concept study.

**Field-collected samples**

No field-collected samples were used in this study.

**Ethics oversight**

Ethical approval of this study was granted by the Home Office of the United Kingdom (PP1309593) and the Animal Welfare and

| Ethics oversight | Ethical Review Body (AWERB) at the Animal and Plant Health Agency (APHA). All animal procedures were carried out in accordance with the United Kingdom Animals (Scientific Procedures) Act of 1986. |
| --- | --- |

Note that full information on the approval of the study protocol must also be provided in the manuscript.

## Plants

| Seed stocks | N/A |
| --- | --- |
| Novel plant genotypes | N/A |
| Authentication | N/A |
